# Incidental parathyroidectomy during thyroid surgery and postoperative hypocalcemia: a multicenter retrospective cohort study

**DOI:** 10.3389/fsurg.2026.1946603

**Published:** 2026-10-14

**Authors:** Nuha Alsaleh, Khadija AlKhawaja, Abdullah S. Al-Darwish, Saad Alshehri, Ammar Alzahim, Osama N. Almubadel, Hala Abu-Qasem, Reema M. Almutairi, Reem AlaaEldeen Ahmed

**Affiliations:** 1 Department of Surgery, College of Medicine, King Saud University, Riyadh, Saudi Arabia; 2 Department of Surgery, Arryan Hospital, Dr. Sulaiman Al Habib Medical Group, Riyadh, Saudi Arabia; 3 Department of Surgery, King AbdulAziz Medical City, National Guard, Riyadh, Saudi Arabia; 4 College of Medicine, Alfaisal University, Riyadh, Saudi Arabia

**Keywords:** endocrine surgery, incidental parathyroidectomy, parathyroid preservation, postoperative hypocalcemia, Saudi Arabia, thyroidectomy

## Abstract

**Background:**

Postoperative hypocalcemia is the most frequent metabolic complication of thyroidectomy, and incidental parathyroidectomy (IPT) is a commonly cited but incompletely characterized contributor. Despite a substantial published literature, the independent clinical impact of IPT on postoperative calcium homeostasis remains debated, and multicenter data from high-volume endocrine surgery settings in Saudi Arabia are lacking.

**Methods:**

A retrospective multicenter cohort study was conducted across high-volume endocrine surgery units in Saudi Arabia between January 2020 and December 2024. A total of 455 patients who underwent total or subtotal thyroidectomy were included. IPT was defined histopathologically. Predictors of IPT and of postoperative hypocalcemia were assessed using pre-specified multivariable logistic regression, with cluster-robust standard errors accounting for the multicenter structure. Adjusted odds ratios (aORs) with 95% confidence intervals are reported.

**Results:**

IPT was confirmed in 145 of 455 patients (31.9%). In a pre-specified multivariable model, age ≥60 years was associated with IPT with conventional standard errors (aOR 1.72, 95% CI 1.04–2.85; *p* = 0.035) but not after accounting for clustering by operating surgeon (aOR 1.72, 95% CI 0.70–4.24; *p* = 0.24); no variable was a robust independent predictor. When postoperative hypocalcemia was modeled directly, IPT was not associated with it (aOR 0.94, 95% CI 0.44–2.00; *p* = 0.87), whereas total thyroidectomy was the dominant predictor (aOR 4.82, 95% CI 1.44–16.07; *p* = 0.011). Preoperative parathyroid hormone was available for only 112 of 455 patients (24.6%) and, using an appropriate non-parametric test, did not differ significantly by IPT status (4.00 ± 1.98 vs. 5.73 ± 4.88 pg/mL; *p* = 0.14, Mann–Whitney U test). IPT was not associated with calcium supplementation requirement or readmission for hypocalcemia.

**Conclusions:**

IPT occurred in nearly one-third of patients but did not predict postoperative hypocalcemia; the extent of surgery, specifically total thyroidectomy, was the principal determinant of postoperative calcium disturbance. Age and preoperative parathyroid hormone were not robust independent correlates once clustering and appropriate testing were applied. These results support universal postoperative calcium monitoring after total thyroidectomy rather than reliance on histologic parathyroid findings.

## 1 Introduction

Thyroidectomy ranks among the most commonly performed endocrine operations worldwide, and its frequency has grown steadily over the past two decades alongside rising detection rates for nodular thyroid disease and differentiated thyroid carcinoma (1, 2). Despite meaningful advances in surgical technique and perioperative care, postoperative hypocalcemia remains the procedure's most prevalent metabolic complication, affecting between 20% and 50% of patients following total thyroidectomy depending on the population studied and the definition applied (3, 4). The burden this complication places on patients makes its prevention and early identification a persistent priority in endocrine surgery.

One contributor to postoperative hypocalcemia that has received comparatively less systematic attention is incidental parathyroidectomy (IPT): the unintentional removal of one or more parathyroid glands within the thyroidectomy specimen. Reported rates vary widely, from as low as 3% to as high as 31% across published series, with this variation largely attributable to differences in surgical extent, underlying thyroid pathology, and the rigor of histopathological examination (5, 6). The clinical concern is straightforward: each inadvertently excised gland reduces total parathyroid reserve, potentially tipping the postoperative balance toward symptomatic calcium deficiency. Yet whether this histologic finding translates into a meaningful and independent effect on clinical outcomes, beyond what is already explained by the extent of surgery, remains genuinely contested (7, 8).

Part of the difficulty lies in the anatomy. The superior parathyroid glands occupy a relatively predictable position near the posterior cricoid cartilage, but the inferior glands are anatomically variable: they may lie in ectopic positions, descend with the thymus, or be embedded within the thyroid parenchyma itself (9, 10). This variability makes inadvertent excision difficult to eliminate entirely, particularly during total thyroidectomy or central neck dissection, where bilateral capsular dissection and vascular stripping of the inferior thyroid artery territory can compromise both gland identification and perfusion (11, 12).

The published evidence on IPT's clinical consequences is divided. Several studies and meta-analyses have identified it as an independent predictor of early biochemical hypocalcemia and increased calcium supplementation requirement (3, 5). Others find no significant association once surgical extent is controlled for, raising the possibility that parathyroid devascularization, which leaves no histologic trace in the specimen, may be the more consequential mechanism (10, 13) Heterogeneous definitions of hypocalcemia, variable histopathological protocols, and substantial differences in institutional volume and technique all contribute to this inconsistency (6, 8).

Within Saudi Arabia, the available data suggest IPT occurs in 16%–23% of thyroidectomy specimens, with higher rates associated with malignant pathology and more extensive surgical procedures (12, 14). These figures come largely from single-center series with variable biochemical follow-up, making it difficult to draw firm conclusions about the true incidence or its independent clinical impact in this population.

This study addresses that gap. Using data from multiple high-volume endocrine surgery units in Saudi Arabia, we aimed to determine the incidence and independent predictors of IPT in a cohort of 455 patients undergoing thyroidectomy, and to evaluate its association with postoperative hypocalcemia, calcium supplementation requirement, and hospital readmission.

## 2 Methods

### 2.1 Study design and setting

This was a retrospective multicenter cohort study evaluating the incidence, predictors, and clinical consequences of incidental parathyroidectomy following thyroid surgery. Data were drawn from high-volume endocrine surgery units in Saudi Arabia, covering the period from January 2020 to December 2024. All included procedures were performed by board-certified endocrine surgeons completing more than 50 thyroidectomies per year. Ethical approval was granted by the Institutional Review Board of King Saud University Medical City (Ref. No. E-25-9837). The study was conducted in accordance with the Declaration of Helsinki. All patient records were anonymized prior to analysis.

### 2.2 Study population and eligibility criteria

Eligible patients were adults aged 18 years or older who underwent total or subtotal thyroidectomy for any benign or malignant thyroid indication during the study period, with complete medical, operative, histopathologic, and biochemical records. Exclusion criteria included revision or reoperative surgery, modified or radical neck dissection, procedures for hyperparathyroidism, incomplete records, and loss to follow-up within six months. After applying these criteria, 455 patients were deemed eligible for final analysis, of whom 145 were found to have incidental parathyroidectomy confirmed on histopathological examination (Figure 1).

**Figure 1:**
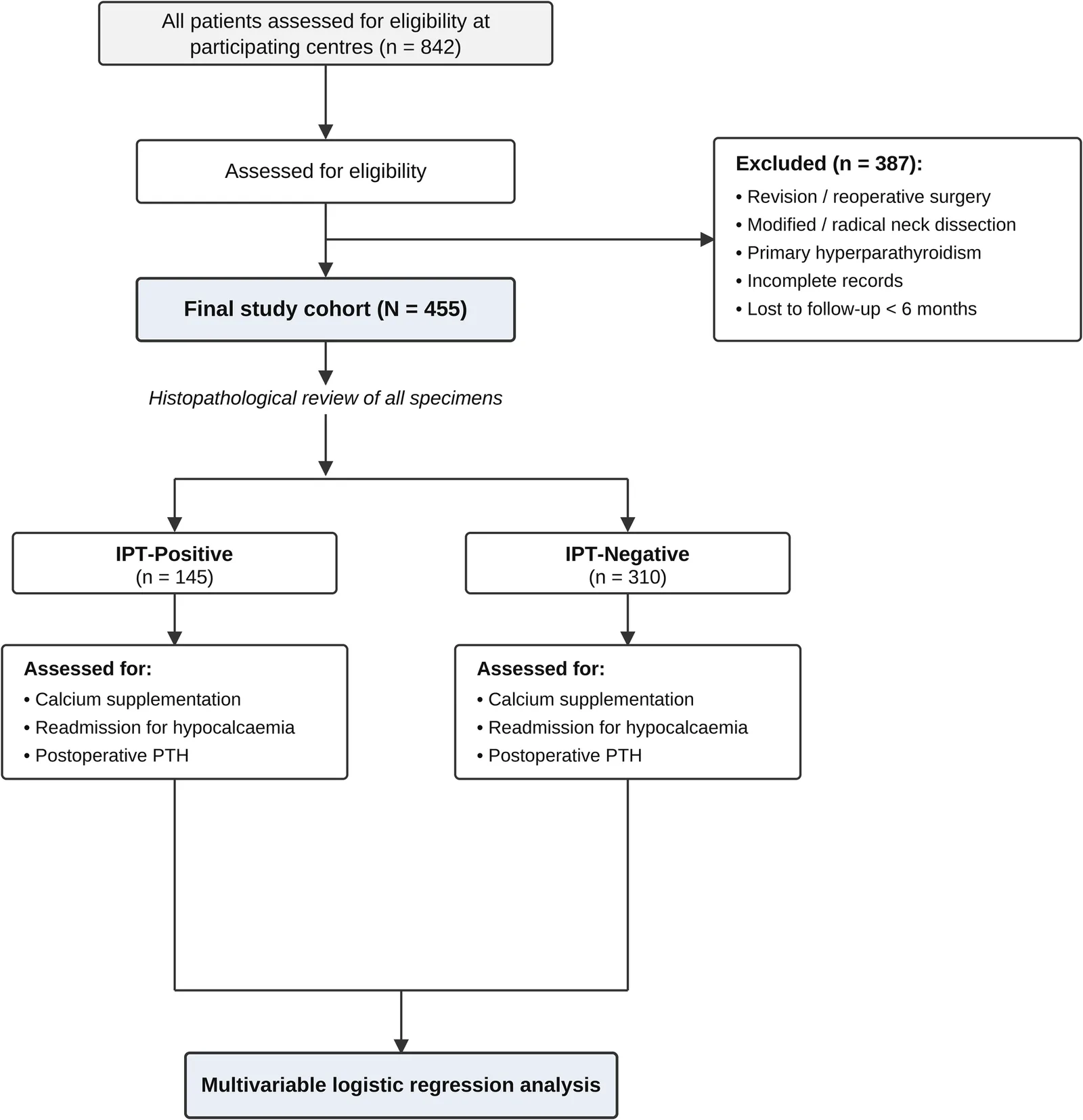
Patient flow diagram. IPT, incidental parathyroidectomy; PTH, parathyroid hormone.

### 2.3 Definitions and outcome measures

Incidental parathyroidectomy was defined as the presence of parathyroid tissue in the thyroidectomy specimen not intentionally removed during surgery (5, 6) Patients were categorized as IPT-positive (≥1 gland identified) or IPT-negative (no parathyroid tissue detected). Postoperative hypocalcemia was defined as a total serum calcium below 2.0 mmol/L (8.0 mg/dL) on the first available postoperative measurement. Serum albumin was not consistently recorded in the source data, so albumin-corrected calcium could not be calculated; uncorrected total calcium was therefore used, and this is acknowledged as a limitation. Transient hypocalcemia: correction within six months. Permanent hypocalcemia: continued replacement beyond six months (4, 15). Secondary Outcomes included calcium supplementation requirement during or after hospitalization; readmission for hypocalcemia; postoperative intact PTH at 24 h.

### 2.4 Data collection

Data were retrieved retrospectively from electronic medical records, operative logs, anesthesia charts, and pathology reports using a standardized extraction sheet across all sites. Double entry was performed for 10% of randomly selected records; discrepancies were resolved by consensus. The extent of missing data was quantified for each variable. Preoperative parathyroid hormone was the most affected, available for 112 of 455 patients (24.6%), and postoperative 24-hour parathyroid hormone for 32 patients. Available-case analysis was used, with the number of patients contributing to each estimate reported alongside the result. Because preoperative parathyroid hormone was substantially incomplete, we examined whether its availability was associated with measured patient characteristics to assess the plausibility of a missing-at-random assumption; availability was higher after total thyroidectomy (29% vs. 17%; *p* = 0.006), indicating the data were not missing completely at random.

### 2.5 Statistical analysis

All analyses were performed using IBM SPSS Statistics version 28.0 and R version 4.3.1. Continuous variables are reported as mean ± standard deviation (SD); categorical variables as *n* (%). Independent t-tests and chi-square or Fisher's exact tests were used for between-group comparisons. Independent predictors of incidental parathyroidectomy were assessed using a single pre-specified multivariable binary logistic regression model with covariates chosen *a priori* (age ≥60 years, sex, preoperative diagnosis, and extent of surgery); no data-driven or stepwise selection was used. Postoperative hypocalcemia was analyzed as a separate pre-specified logistic model with incidental parathyroidectomy status, extent of surgery, and age among the covariates. To account for the multicenter structure, the operating surgeon was used as the clustering unit, and cluster-robust standard errors were computed; because the number of surgeons was small, these are reported as a sensitivity analysis alongside the conventional estimates. Adjusted odds ratios (aORs) with 95% confidence intervals (CIs) are reported throughout. Model fit was evaluated by the Hosmer-Lemeshow test and Nagelkerke's R^2^. All tests were two-tailed; *p* < 0.05 was considered statistically significant. Because preoperative parathyroid hormone was markedly skewed, group comparisons for this variable used the Mann–Whitney U test.

## 3 Results

### 3.1 Study cohort characteristics

A total of 455 patients were included; IPT was confirmed in 145 (31.9%). The cohort was predominantly female (83.5%), with a mean age of 44.96 ± 14.43 years. The most common diagnosis was thyroid malignancy (49.9%), followed by multinodular goiter (45.9%). Total thyroidectomy was performed in 65.7%.

### 3.2 Comparison of IPT-positive and IPT-negative groups

As shown in Table 1, the two groups were broadly comparable across baseline characteristics. IPT-positive patients were slightly older (46.39 ± 13.61 vs. 44.29 ± 14.77 years, *p* = 0.149) and more likely to have undergone total thyroidectomy (71.7% vs. 62.9%, *p* = 0.082), though neither difference was statistically significant. Preoperative PTH was numerically lower in IPT-positive patients (4.00 ± 1.98 vs. 5.73 ± 4.88 pg/mL; *n* = 112), but the difference was not statistically significant using the Mann–Whitney U test appropriate for its skewed distribution (*p* = 0.14), and preoperative PTH was available for only a quarter of the cohort. Postoperative calcium, supplementation rates, and readmission rates were comparable (Figure 2).

**Table 1:** Baseline demographic, clinical, biochemical, surgical, and pathological characteristics of the study cohort by IPT Status (*N* = 455).

Variable	Total (*N* = 455)	IPT-Positive (*n* = 145)	IPT-Negative (*n* = 310)	*p*-value
Age (years), mean ± SD				
	44.96 ± 14.43	46.39 ± 13.61	44.29 ± 14.77	0.149
Age category, *n* (%)				
< 30 years	71 (15.6)	18 (12.4)	53 (17.1)	
30–39 years	85 (18.7)	25 (17.2)	60 (19.4)	
40–49 years	132 (29.0)	48 (33.1)	84 (27.1)	
50–59 years	88 (19.3)	21 (14.5)	67 (21.6)	
≥ 60 years	79 (17.4)	33 (22.8)	46 (14.8)	0.052
Sex, *n* (%)				
Female	380 (83.5)	119 (82.1)	261 (84.2)	0.665
Male	75 (16.5)	26 (17.9)	49 (15.8)	
Comorbidities, *n* (%)				
Hypertension	102 (22.4)	35 (24.1)	67 (21.6)	0.630
Diabetes mellitus	77 (16.9)	25 (17.2)	52 (16.8)	1.000
Multiple comorbidities	174 (38.2)	55 (37.9)	119 (38.4)	1.000
Preoperative diagnosis, *n* (%)				
Malignant	278 (61.1)	92 (63.4)	186 (60.0)	0.549
Benign	177 (38.9)	53 (36.6)	124 (40.0)	
Type of surgery, *n* (%)				
Total thyroidectomy	299 (65.7)	104 (71.7)	195 (62.9)	0.082
Subtotal thyroidectomy	156 (34.3)	41 (28.3)	115 (37.1)	
Thyroid pathology, *n* (%)				
Thyroid malignancy	227 (49.9)	67 (46.2)	160 (51.6)	0.444
Multinodular goiter	209 (45.9)	71 (49.0)	138 (44.5)	
Hashimoto's thyroiditis	16 (3.5)	5 (3.4)	11 (3.5)	
Graves’ disease	3 (0.7)	2 (1.4)	1 (0.3)	
Preoperative biochemistry, mean ± SD				
Total calcium (mmol/L)	2.26 ± 0.15 (*n* = 422)	2.27 ± 0.12 (*n* = 130)	2.26 ± 0.16 (*n* = 292)	0.511
Phosphorus (mmol/L)	1.15 ± 0.23 (*n* = 447)	1.17 ± 0.26 (*n* = 141)	1.15 ± 0.22 (*n* = 306)	0.336
PTH preop (pg/mL) †	5.13 ± 4.18 (*n* = 112)	4.00 ± 1.98 (*n* = 39)	5.73 ± 4.88 (*n* = 73)	0.14
Postoperative biochemistry, mean ± SD				
Calcium postop (mmol/L)	2.18 ± 0.22 (*n* = 400)	2.17 ± 0.31 (*n* = 124)	2.18 ± 0.16 (*n* = 276)	0.800
PTH 24 h (pg/mL)	4.80 ± 5.06 (*n* = 32)	4.45 ± 2.37 (*n* = 11)	4.99 ± 6.07 (*n* = 21)	0.780
Postoperative outcomes, *n* (%)				
Calcium supplementation	268 (58.9)	81 (55.9)	187 (60.3)	0.424
Readmission for hypocalcemia	11 (2.4)	3 (2.1)	8 (2.6)	0.997

Preoperative PTH comparison uses the Mann–Whitney U test appropriate for its skewed distribution and was not statistically significant; PTH was available for 112 patients only. Other *p*-values from independent t-test (continuous) or chi-square/Fisher exact test (categorical). IPT, incidental parathyroidectomy; PTH, parathyroid hormone.

**Figure 2:**
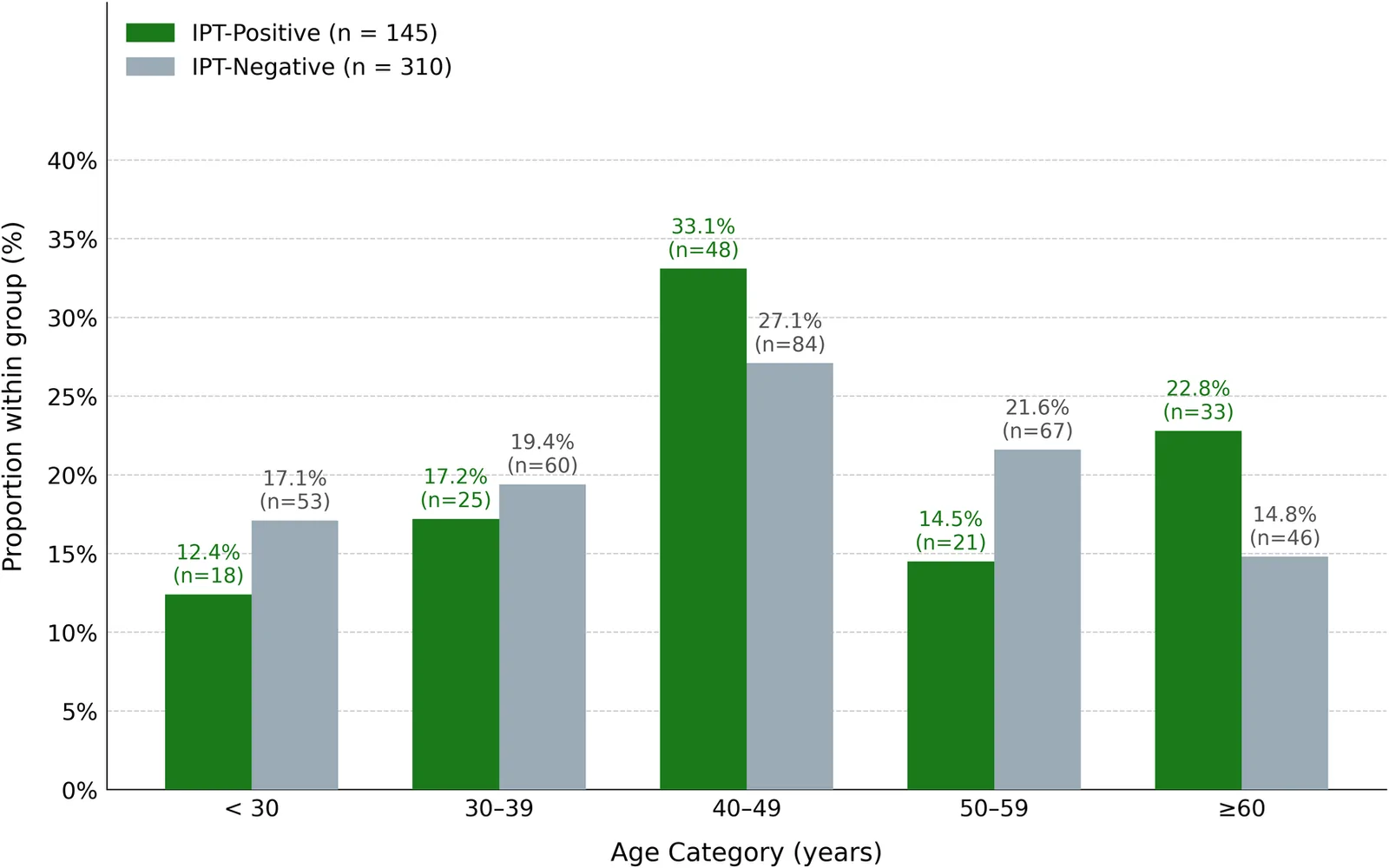
Age category distribution by IPT Status. IPT, incidental parathyroidectomy. *p* = 0.052 for overall age distribution comparison between groups (chi-square test).

### 3.3 Independent predictors of incidental parathyroidectomy

In the pre-specified multivariable model (Table 2), age ≥60 years was associated with IPT using conventional standard errors (aOR 1.72, 95% CI 1.04–2.85; *p* = 0.035), and total thyroidectomy showed a borderline association (aOR 1.46, 95% CI 0.94–2.27; *p* = 0.090); sex and malignant diagnosis were not associated. The overall model was not statistically significant (likelihood-ratio *p* = 0.080; Nagelkerke R^2^ = 0.015). When standard errors were clustered by operating surgeon to account for the multicenter structure, the age association was attenuated and no longer significant (aOR 1.72, 95% CI 0.70–4.24; *p* = 0.24). No variable was a robust independent predictor of IPT (Figure 3).

**Table 2:** Pre-specified multivariable logistic regression for independent predictors of incidental parathyroidectomy (*N* = 455).

Predictor	aOR (95% CI), standard	aOR (95% CI), cluster-robust^a^	p^b^
Age ≥60 years	1.72 (1.04–2.85)	1.72 (0.7–4.24)	0.035
Female sex	0.83 (0.49–1.41)	0.83 (0.57–1.22)	0.496
Malignant diagnosis	1.13 (0.74–1.73)	1.13 (0.72–1.78)	0.564
Total thyroidectomy	1.46 (0.94–2.27)	1.46 (0.45–4.71)	0.090

Reference groups: age <60 years; male sex; benign diagnosis; subtotal thyroidectomy. Model: *n* = 455, df = 4, likelihood-ratio *p* = 0.080, Nagelkerke pseudo-R^2^ = 0.015.

a Cluster-robust standard errors by operating surgeon.

b p from the standard model. Age ≥60 years is significant with standard errors but not after accounting for surgeon clustering.

**Figure 3:**
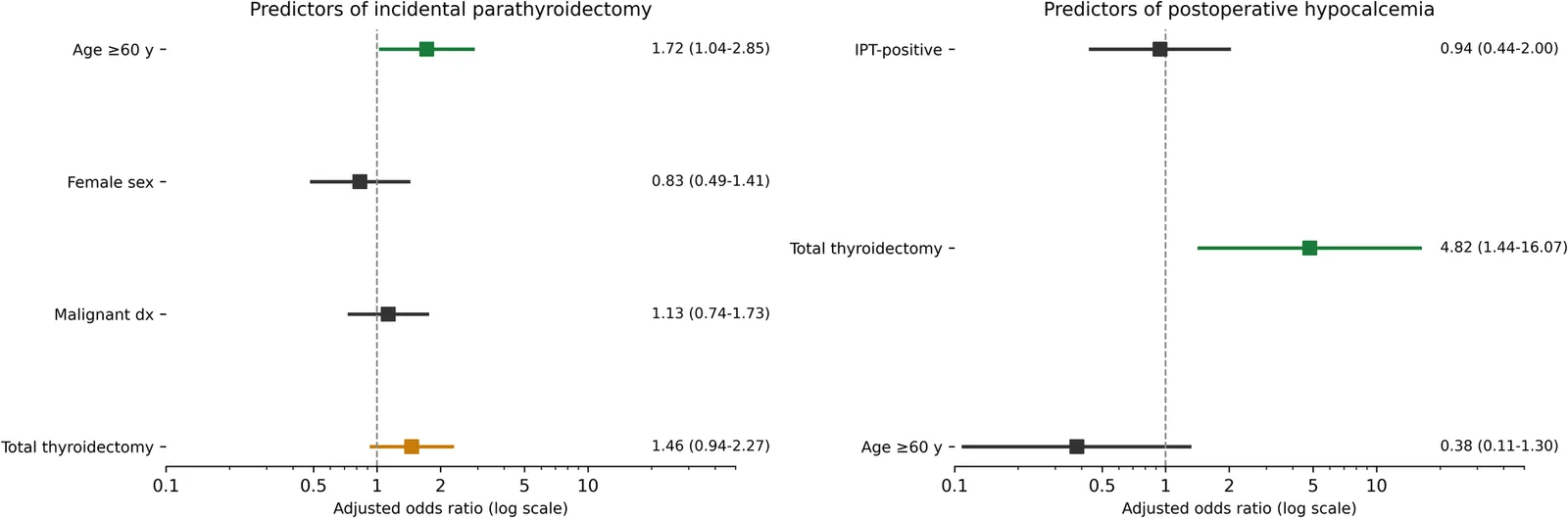
Forest plots of adjusted odds ratios (aOR) with 95% confidence intervals from the two pre-specified multivariable logistic regression models. Left panel: predictors of incidental parathyroidectomy (age ≥60 years, female sex, malignant diagnosis, total thyroidectomy). Right panel: predictors of postoperative hypocalcemia (incidental parathyroidectomy, total thyroidectomy, age ≥60 years). Squares are point estimates and horizontal lines the 95% CI; the dashed vertical line marks aOR = 1 (no effect). The x-axis is on a logarithmic scale. IPT, incidental parathyroidectomy.

### 3.4 Direct analysis of hypocalcemia

In the multivariable model with postoperative hypocalcemia (total calcium < 2.0 mmol/L) as the dependent variable, based on 400 patients with a recorded postoperative calcium and 37 events, IPT was not associated with hypocalcemia (aOR 0.94, 95% CI 0.44–2.00; *p* = 0.87). Total thyroidectomy was the dominant predictor (aOR 4.82, 95% CI 1.44–16.07; *p* = 0.011), a finding that strengthened under surgeon-clustered estimation (Table 3, Figure 3). Unadjusted outcomes were consistent with this pattern (Table 4): hypocalcemia occurred in 11 of 124 IPT-positive vs. 26 of 276 IPT-negative patients (8.9% vs. 9.4%), calcium supplementation in 55.9% vs. 60.3%, and readmission for hypocalcemia in 2.1% vs. 2.6%; none differed significantly by IPT status.

**Table 3:** Multivariable logistic regression for postoperative hypocalcemia (*n* = 400; 37 events).

Predictor	aOR (95% CI), standard	aOR (95% CI), cluster-robust^a^	p
Incidental parathyroidectomy	0.94 (0.44–2)	0.94 (0.32–2.72)	0.871
Total thyroidectomy	4.82 (1.44–16.07)	4.82 (2.28–10.19)	0.011
Age ≥60 years	0.38 (0.11–1.3)	0.38 (0.22–0.67)	0.125

Outcome: postoperative total calcium < 2.0 mmol/L (uncorrected; serum albumin was not recorded, so albumin correction could not be applied). Model: *n* = 400, events=37, likelihood-ratio *p* = 0.004. Incidental parathyroidectomy was not associated with hypocalcemia; total thyroidectomy was the dominant predictor.

a Cluster-robust standard errors by operating surgeon.

**Table 4:** Postoperative outcomes by incidental parathyroidectomy status (unadjusted).

Outcome	IPT-positive (*n* = 145)	IPT-negative (*n* = 310)	p
Hypocalcemia, n/N (%)	11/124 (8.9)	26/276 (9.4)	0.87
Calcium supplementation, *n* (%)	81 (55.9)	187 (60.3)	0.42
Readmission for hypocalcemia, *n* (%)	3 (2.1)	8 (2.6)	0.74

Hypocalcemia=postoperative total calcium < 2.0 mmol/L among patients with a recorded value. Adjusted estimates are in Table 3.

## 4 Discussion

This multicenter retrospective cohort of 455 patients undergoing thyroid surgery in Saudi Arabia found an incidental parathyroidectomy rate of 31.9%, with no variable serving as a robust independent predictor of IPT once the multicenter structure of the data was accounted for. When postoperative hypocalcemia was modeled directly, IPT was not associated with it, whereas total thyroidectomy was the dominant predictor, reinforcing that the extent of surgery rather than the histologic finding of parathyroid tissue governs postoperative calcium disturbance (3, 5). These findings do not diminish the clinical importance of parathyroid preservation, but they suggest that histologic IPT, on its own, may not be the most reliable marker of functional parathyroid compromise in the postoperative period.

The observed IPT rate of 31.9% sits at the upper boundary of the range reported in the international literature, which spans from approximately 3% to 31% (6–8). This is broadly consistent with cohorts in which total thyroidectomy predominates and cancer-related procedures constitute a substantial proportion of cases. The (7) single-center Italian study of 799 patients reported an IPT rate of 21.9%, with younger age and concurrent neck dissection as principal risk factors, a contrast with the older-age association we observed, likely reflecting differences in population demographics and surgical mix.

Although age ≥60 years was not a robust independent predictor once clustering was accounted for, the modest age signal observed with conventional standard errors is biologically plausible. Older patients tend to present with more anatomically complex thyroid disease, more fibrotic perithyroidal tissue, and a higher prevalence of intrathyroidal parathyroid gland positioning, all of which increase the likelihood of inadvertent excision (9, 11).

Total thyroidectomy showed a consistent borderline association with IPT across both models, aligning with the mechanistic understanding that bilateral procedures carry greater parathyroid risk through capsular dissection along both thyroid lobes and vascular compromise of the inferior parathyroid blood supply (11–13). That this fell short of significance likely reflects moderate sample size and the absence of central neck dissection data.

The lower preoperative PTH in IPT-positive patients (4.00 ± 1.98 vs. 5.73 ± 4.88 pg/mL, *p* = 0.036) has not been prominently reported in prior IPT literature, and it should be interpreted cautiously for two reasons. First, preoperative PTH was available for only 112 of 455 patients (24.6%), so the comparison rests on a minority of the cohort, and its availability was higher after total thyroidectomy, indicating the data were not missing at random. Second, using the Mann–Whitney U test appropriate for its skewed distribution, the difference was not statistically significant (*p* = 0.14). Circulating PTH is in any case a noisy marker of parathyroid reserve, secreted in pulsatile fashion with substantial biological variability, so a single preoperative value may not reflect the number or functional capacity of at-risk glands. This limitation is echoed in the adjacent parathyroid literature, where preoperative PTH showed no significant correlation with parathyroid adenoma volume, a dissociation attributed to the pulsatile secretion and variability of PTH rather than a true absence of relationship (16). Whether reduced baseline parathyroid reserve predisposes to inadvertent excision, or whether this reflects incomplete and non-random ascertainment, cannot be resolved retrospectively and warrants prospective study.

The null finding on hypocalcemia outcomes is consistent with several single-institution studies (10, 13, 17). The most compelling explanation is that surgical extent, and the parathyroid devascularization that accompanies extensive capsular dissection, is the dominant determinant of postoperative calcium disruption in this cohort. Devascularization leaves no histologic trace yet can render remaining *in-situ* glands functionally inadequate, meaning IPT-negative patients may still experience significant parathyroid impairment (15, 18).

Prior Saudi studies have reported IPT rates of 16%–23%, with transient hypocalcemia as the predominant consequence (12, 14, 19). Our multicenter data extend this evidence base, reinforcing that structured perioperative calcium monitoring, applied universally after total thyroidectomy regardless of intraoperative parathyroid findings, remains the more defensible standard of care (4).

Near-infrared autofluorescence (*N*IRAF) imaging has emerged as a promising adjunct for real-time parathyroid identification, with multicenter data suggesting it reduces rates of incidental parathyroid tissue in final pathology specimens (20–22). Our findings, which identify patients undergoing total thyroidectomy as the group in whom postoperative calcium disturbance concentrates, define a clinically identifiable high-risk subgroup in whom targeted adoption of NIRAF is most justified.

Strengths include multicenter design, histopathologic confirmation of all IPT cases, standardized operative protocols. Limitations include retrospective design, absence of central neck dissection data, and the incomplete 24-hour postoperative PTH datasets analyzed on an available-case basis. Serum albumin was not consistently recorded, so hypocalcemia was defined using uncorrected total calcium. The multicenter structure was represented by a small number of operating surgeons, which limits formal multilevel modeling and means the clustering analysis should be read as a sensitivity analysis. Power for the permanent hypocalcemia subgroup was limited.

## 5 Conclusion

Incidental parathyroidectomy occurred in nearly one-third of patients in this multicenter Saudi cohort but was not associated with postoperative hypocalcemia, calcium supplementation, or readmission. The extent of surgery, specifically total thyroidectomy, was the principal determinant of postoperative calcium disturbance, whereas neither age nor preoperative parathyroid hormone was a robust independent correlate once clustering and appropriate statistical testing were applied. These results support universal postoperative calcium monitoring after total thyroidectomy rather than reliance on intraoperative or histologic parathyroid findings, and the targeted deployment of parathyroid-identification technologies in patients undergoing more extensive surgery.

## Data Availability

The original contributions presented in the study are included in the article/Supplementary Material, further inquiries can be directed to the corresponding author.
